# Two fluid model in low energy excited states within spin-ice systems

**DOI:** 10.1038/s41598-018-34529-x

**Published:** 2018-11-02

**Authors:** F. I. López-Bara, F. López-Aguilar

**Affiliations:** Electromagnetism Group, Department of Physics, Autonomous Barcelona University, Bellaterra, E-08193 Barcelona Spain

## Abstract

Excitations in magnetic structures of the so-called spin-ice materials generate two different peaks in the specific heat and anomalies in entropy in the temperature interval between 0 and 1 K. These points are due to the existence of two low-energy excited global states which seem to transit from a bosonic condensate towards a magnetic neutral plasma in a narrow temperature interval between 0.05 ≤ T ≤ 1 K. In this paper, we determine the characteristic features of two states and we analyze the possibilities of existence of a BEC state and its phase transition to the magnetic plasma state from a model of two magnetic charge fluids. From the structural analysis of the many-body excitation states, we obtain theoretical results about entropy and specific heat since these two key physical magnitudes announce the phase transitions. We give criteria for distinguishing if some of these phase transitions is of either first or second order.

## Introduction

The so-called “spin-ices”^1–4^, the topological insulators^5^ and the real magnets synthesized^6^ can present excited low energy states represented in modifications in the magnetic structures that can be interpreted as entities whose behavior is similar to magnetic charges^1–4^. The properties that present these modifications of their magnetic structures could have a double interest: on one hand, it could allow us to discover new characteristics for low-energy excitation states within the magnetic theory and on the other hand, could imply that these compounds were serious candidates for devices capable of propagating both information and energy^3,7,8^. The compounds of Holmium or Disprosium Dy_2_Ti_2_O_7_ and Dy_2_Ti_2_O_7_, which crystallize in a pyrochlore type [refs^3,4^ and references therein] present a magnetic structure whose main characteristic is that spin-flips in the magnetic moments of the lanthanide ions placed in the vertexes shared by contiguous tetrahedrons can progress in their crystal structures. Bramwell *et al*.^3^ analyzed in 2009 the mobility of these modifications of their magnetic structure under the presence of external magnetic fields and whose effects were coined as magnetricity. The fundamental ground many-body state of the magnetic structure consists of tetrahedrons connected by a vertex in which there is a magnetic ion whose magnetic moment is shared by two connected tetrahedronsIn this situation, if in one of the tetrahedron the magnetic moment points to the center of the tetrahedron, in its contiguous one, obviously, points in the opposite direction.

This situation is identical for each vertex of each tetrahedron. In this paper, we analyze a formalized theory of the spin state of each tetrahedron as well as the global many-particle state. Each tetrahedron state can be represented by the following schematic form $|t\rangle =|\uparrow ,\uparrow ,\downarrow ,\downarrow \rangle$. Where with the upward arrows we want to indicate that the magnetic moment has an exit direction of the tetrahedron and with the arrows in the opposite direction we indicate that the corresponding magnetic moments point towards the direction of the center of the tetrahedron. Therefore, all magnetic moments in the fundamental state of the global system have the direction pointing to the barycenter of each tetrahedron, two in outward direction of the tetrahedron and the other two in inward direction.

Consequently, the fundamental ground many-body state of the magnetic structure consists of tetrahedrons connected by a vertex in which there is a magnetic ion whose magnetic moment is shared by two connected tetrahedrons. This situation is identical for each vertex of each tetrahedron. The dumbbell model^1,2^ associates at each magnetic moment the existence of two virtual magnetic charges, ${{\rm{q}}}_{{\rm{m}}}=\pm \frac{|\overrightarrow{{\rm{\mu }}}|}{{\rm{l}}}$, a negative charge at the origin of the magnetic moment vector $\overrightarrow{{\rm{\mu }}}$ of the Lanthanide atom, and a positive one at the end of that vector, in a dual image analogous to the electrical dipole momentum. Therefore each tetrahedron in the ground state of the system contains two negative, $-\frac{|\overrightarrow{{\rm{\mu }}}|}{{\rm{l}}}$ charges and two positive ones, $\frac{|\overrightarrow{{\rm{\mu }}}|}{{\rm{l}}}$. When the temperature increases slightly there is a probability that a magnetic moment of a vertex undergoes a change of direction of its magnetic moment. This fact in the dumbbell model would imply that one of the connected tetrahedrons should have three positive magnetic charges and one negative and the corresponding connected tetrahedron should have three negative magnetic charges and one positive. This would lead us to the effective image that for each pair of tetrahedra in which there was an inversion of the magnetic moment in a shared vertex, in a tetrahedron of the pair there is a magnetic charge, g = 2|q_m_|, constituted of two original virtual charges, in the another one there is a negative magnetic charge, g = −2|q_m_|.

These resulting magnetic charges are those that Bramwell *et al*.^3,4^ have discovered that can be moved inside the magnetic structure when the inversion of the magnetic moment travels over the magnetic structure. Therefore, the fundamental state is formed by $|{{\rm{\Phi }}}_{0}\rangle =\prod _{{\rm{i}}}^{{\rm{N}}}|{{\rm{t}}}_{{\rm{i}}},\,{{\rm{t}}^{\prime} }_{{\rm{i}}}\rangle$, where i-index runs over al vertexes of the tetrahedrons of the crystal; |t_i_〉 is the tetrahedron in which the magnetic moment enters its interior and $|{{\rm{t}}^{\prime} }_{{\rm{i}}}\rangle$ is the tetrahedron in which the magnetic moment points outside of the tetrahedron.

## The Lowest-Energy Excitation State as Independent Magnetic Dipoles (Without Considering Inter-Dipolar Interaction)

The spin-flips among the pairs of contiguous tetrahedrons occur with a reduced energy expenditure. When they run over through the crystal, modifying the local magnetic moment of each tetrahedron, Castelnovo *et al*.’s model^1^ interpreted as resulting interacting magnetic charges which form weak crystalline structures that have similarities with the Dirac strings^1–3^. The elemental excitation state can be analytically described by the expression ${{\rm{S}}}_{{\rm{i}}}^{-}|{{\rm{t}}}_{{\rm{i}}},\,{{\rm{t}}^{\prime} }_{i}\rangle$, where ${{\rm{S}}}_{{\rm{i}}}^{-}={{\rm{S}}}_{{\rm{i}}}^{{\rm{x}}}-{{\rm{jS}}}_{{\rm{i}}}^{{\rm{y}}}$, with ${{\rm{S}}}_{{\rm{i}}}^{{\rm{x}}}$(${{\rm{S}}}_{{\rm{i}}}^{{\rm{y}}}$) being the x(y) components of the $\overrightarrow{{\rm{S}}}$-spin of i-atom located in the shared vertex of two tetrahedrons of each pair. When the spin-flips travel within the crystal, then the length of these strings increases and in the limit, the resulting magnetic monopoles can be considered quasi-free magnetic charges^1,3,4^. Thus, the state ${{\rm{S}}}_{{\rm{i}}}^{-}|{{\rm{t}}}_{{\rm{i}}},\,{{\rm{t}}^{\prime} }_{{\rm{i}}}\rangle$ can be written as:

1 $${{\rm{S}}}_{{\rm{i}}}^{-}{|{{\rm{t}}}_{{\rm{i}}},{\rm{t}}^{\prime} \rangle }_{{\rm{i}}}=|{\rm{p}}\rangle \times |{\rm{a}}\rangle =|{\rm{p}},\,{\rm{a}}\rangle $$

where |p〉 (|a〉) is the definition of a positive (negative) magnetic charge whose absolute value is ${\rm{g}}=\mathrm{2|}\overrightarrow{{\rm{\mu }}}|/{\rm{L}},$ where L is the length separation between the two centers of the corresponding tetrahedrons of the pair. These structural entities require the existence of a magnetic structure such as that of the spin-ice compounds and they are different from the magnetic monopoles whose existence the high energy physics researchers justified eight decades ago^4,5^ and several laboratories and scientists have tried insistently to find (see for instance, refs^9–15^).

The formation magnetic dipoles according to the dumbbell model implies that the nature of one-body components of the many-body state can be bosonic since the simplest components are constituted of two magnetic charges in a confined and composed particle. The first global state of the spin-ice system corresponds to the highest peak of specific heat. These peaks can be identified and explained by the presence of a pole-antipole pair condensation^1^. This state presents similarities with those situations in which particle-antiparticles are coupled forming composite entities which travel within the many-body system.

One of them is a determined mesonic soup^16^ in which each particle is constituted with a quark and the corresponding antiquark, other different quark-antiquark meson combinations present sensible differences from a pole-antipole pair state. However, the most parallel many-body system with this state which is object of our study is the electron-hole condensate, i.e. the so called electron-hole condensation (exciton condensation) which can appear in the semimetals^17–19^. This requires the fermionic proposal for magnetic monopoles whose nature arises from previous theoretical and experimental analysis and we think that it requires explanation. One of the first and most interesting analysis in this sense emerged in 1997 with the comparison made by Witten^16^ between the quark-antiquark coupling inside hadrons with that formed by the magnetic poles of a dipole and its consequent definition of pole-antipole coupling. More recently, the magnetic monopoles had assigned directly a character of fermionic entities which are empirically detected^20^. In this direction combining the ideas of the authors of refs^16–20^, we have explored the possibility of applying mesonic and excitonic coupling to the dipoles of the dumbbell model^1^.

First of all, we go to analise the highest and narrow peaks of the specific heat which is due to the existence of a large increase of individual components of the many-body state which in this case according to the dumbbell model are confined dipoles. Using the Classical Statistic Physics, in Eq. (4) of the supplementary material of this paper, we determine the potential energy due to the existence of the other magnetic charges. Therefore, the energy for any charge is equal to its potential energy plus the energy of its own spin flip which generates the mentioned magnetic charge. In similar way we determine the corresponding energy of the confined dipoles. Consequently, the energy for a charge is:

2 $${\rm{e}}={{\rm{K}}}_{{\rm{B}}}{\rm{Tln}}\frac{{{\rm{n}}}_{0}}{{\rm{n}}(\overrightarrow{{\rm{r}}},\,{\rm{T}})}+\frac{{\rm{T}}}{{{\rm{T}}}_{{\rm{V}}}}|{\rm{g}}|{{\rm{\varphi }}}_{0}+{{\rm{\Sigma }}}_{0}$$

where Σ_0_ is the self energy (concept introduced in the ref.^1^) of a magnetic charge without movement and the energy of a static confined dipole is

3 $${\rm{e}}^{\prime} =2{{\rm{K}}}_{{\rm{B}}}{\rm{Tln}}\frac{{{\rm{n}}}_{0}}{{\rm{n}}(\overleftarrow{{\rm{r}}},\,{\rm{T}})}+\frac{2{\rm{T}}}{{{\rm{T}}}_{{\rm{V}}}}|{\rm{g}}|{{\rm{\varphi }}}_{0}+{\rm{\Sigma }}{}_{0}-\frac{{{\rm{\mu }}}_{0}{{\rm{g}}}^{2}}{4{\rm{\pi }}d}$$

where the latter term is the attractive interaction between the components of the dipole; d being the splitting betweeen the two charges of the confined dipole which can be estimated as the distance between the two barycentres of the two contiguous tetrahedra.

Having in mind that the dipoles are the coupling of two fermions, we have that the number of magnetic dipoles to any temperature can be determined by

4 $${\rm{M}}=\sum _{{\rm{i}}}\frac{1}{\exp [{\rm{\beta }}({{\rm{e}}^{\prime} }_{{\rm{i}}}-{\rm{\mu }})]-1}$$

To extend to the continuum and using Eq. (3), we obtain:

5 $${\rm{M}}=\frac{{\rm{N}}^{\prime} }{{{\rm{n}}}_{0}}{\int }_{0}^{{{\rm{n}}}_{0}}\frac{{\rm{dn}}}{{(\frac{{{\rm{n}}}_{0}}{{\rm{n}}})}^{2}{{\rm{e}}}^{2{\rm{a}}}{{\rm{e}}}^{{\rm{b}}{\rm{\beta }}}{{\rm{e}}}^{-{\rm{\mu }}{\rm{\beta }}}-1}=\frac{{\rm{N}}}{7{{\rm{n}}}_{0}}{\int }_{0}^{{{\rm{n}}}_{0}}\frac{{{\rm{n}}}^{2}{\rm{zdn}}}{{{\rm{n}}}_{0}^{2}{{\rm{e}}}^{2{\rm{a}}}\,{{\rm{e}}}^{{\rm{b}}{\rm{\beta }}}-{{\rm{n}}}^{2}{\rm{z}}}$$

where ${\rm{a}}=\frac{|{\rm{g}}|{{\rm{\varphi }}}_{0}}{{{\rm{K}}}_{{\rm{B}}}{{\rm{T}}}_{{\rm{V}}}}$; ${\rm{b}}={\rm{\Sigma }}{}_{0}-\frac{{{\rm{\mu }}}_{0}{{\rm{g}}}^{2}}{4{\rm{\pi }}d}$ and ${\rm{\beta }}=\frac{1}{{{\rm{K}}}_{{\rm{B}}}{\rm{T}}}$, ${\rm{z}}={{\rm{e}}}^{{\rm{\mu }}{\rm{\beta }}}$ is the thermodynamic fugacity, M is the number of confined magnetic dipoles, N′ the number of total posible spin flips mainaining the idea of one spin-flip per each pair of tetrahedra and N is the total number of all vertices. In the next section, we explain the factor 1/7 that comes from the exclusion of the possibility of having two spin-flips within any tetrahedron, since then they would have two charges of the same sign whose high (repulsive) positive energy is prohibited or they would be of different sign and as a consequence the spin-flips would be neutralized. Consequently, in each pair of contiguous tetrahedra only one of the seven vertices can present a spin-flip. On the contrary, the energy needed for these situations would be excessive. Using the variable change, x = n^2^z, one can easily obtain the density of:number of confined magnetic monopoles.

6 $${\rm{n}}=\frac{{\rm{M}}}{{\rm{N}}}=\frac{1}{7{{\rm{n}}}_{0}}{\int }_{0}^{{{\rm{n}}}_{0}}\frac{{{\rm{n}}}^{2}{\rm{zdn}}}{{{\rm{n}}}_{0}^{2}{{\rm{e}}}^{2{\rm{a}}}{{\rm{e}}}^{{\rm{b}}{\rm{\beta }}}-{{\rm{n}}}^{2}{\rm{z}}}=\frac{1}{14}\sum _{{\rm{p}}=0}^{\infty }\frac{{{\rm{z}}}^{{\rm{p}}+1}}{({\rm{p}}+\frac{3}{2})\exp [(2{\rm{a}}+{\rm{b}}\,{\rm{\beta }})({\rm{p}}+1)]}=\frac{1}{14}{\rm{S}}({\rm{z}})$$

**Figure 1:**
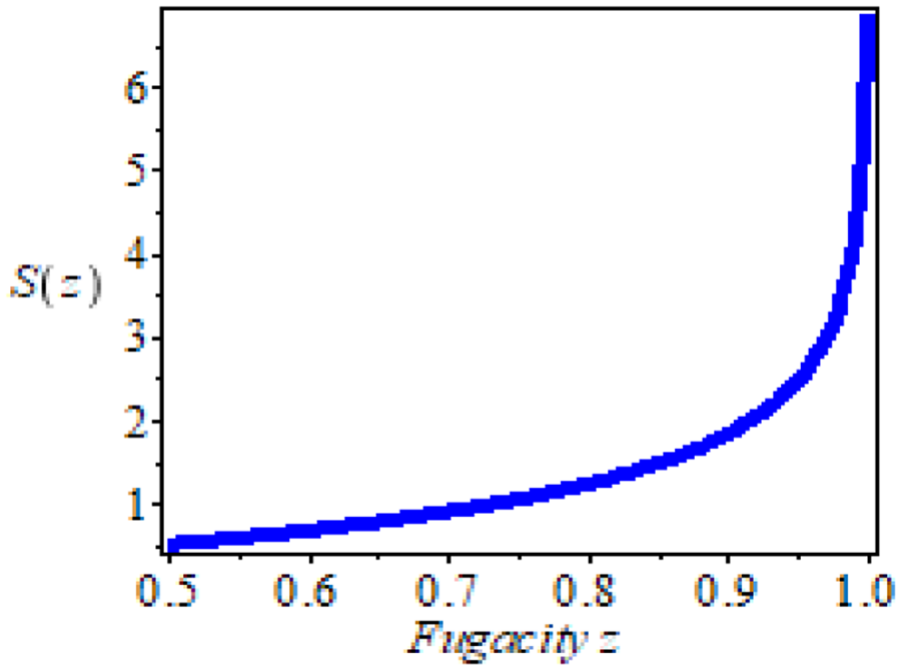
In this plot, we represent the series S(z) of Eq. (6) varying the fugacity z. The convergence of the series is clear and also the divergence of its derivative with respect to z. The divergence of the series of Eq. (7) is a sufficient condition for the existence of a Bose-Einstein condensation if and only if the energy of the individual bosons are in the zero energy as it occurs when the Eq. (8) is accomplished.

The convergence of this serie S(z) of Eq. (6) depends on 2a + bβ and the values of z. The calculation of this series in z is necessary since it is responsible of the existence of a possible boson condensaton when n = n_0_. In Fig. 1, we represent S(z) when 2a + bβ(T_v_) ≅ 0 and as one can see in this Fig. 1 for z = 1 S(z) is around 7.4. The standard criterium for which a boson condensation can be considered as a Bose-Einstein condensate is that practically all individual components of the macroscopic quantum state are in the lowest energy. This criterium is formalized by means of the condition $\frac{{\rm{dn}}}{{\rm{dz}}}\to \infty$ ^21^, i.e. the variation of the density of one body bosons respect to fugacity, z, tends to infinite such as it is shown in Fig. 1. Evidently,

7 $$\frac{{\rm{dn}}}{{\rm{dz}}}=\frac{1}{14}\sum _{{\rm{p}}=0}^{\infty }\frac{({\rm{p}}+1){{\rm{z}}}^{p}}{({\rm{p}}+\frac{3}{2})\exp [(2{\rm{a}}+{\rm{b}}\,{\rm{\beta }})({\rm{p}}+1)]}\mathop{\longrightarrow }\limits^{{\rm{z}}\to 1}\infty $$

The condition (7) is clearly satisfied when 2a + bβ(T_v_) ≅ 0, where T_V_ is the temperature at which there is the maximum in the specific heat this latter condition can also be expressed as

8 $$2|{\rm{g}}|{\varphi }_{0}+{\rm{\Sigma }}{}_{0}-\frac{{{\rm{\mu }}}_{0}{{\rm{g}}}^{2}}{4{\rm{\pi }}\,{\rm{d}}}\cong 0,$$

which implies that the energy of individual bosons is null acording to Eq. (3). With these conditions, for T = T_V_, z = 1, and all magnetic dipoles in the lowest energy state [see Eq. (3)], the boson condensate satisfy the Bose-Einstein condensation.

On the other hand, it must be remembered that the condition 2a + bβ(T_v_) ≅ 0 is sufficient but not necessary for the acomplishement of divergence of Eq. (7), since for small values of 2a + bβ(T_V_), Eq. (7) can also imply $({\rm{N}}^{\prime} \frac{{\rm{dn}}}{{\rm{dz}}}\mathop{\longrightarrow }\limits^{{\rm{z}}\to 1}\infty )$.

## The Lowest Energy State by Considering a First Order Perturbation Interacting System

In section 2, the system is constructed by independent bosons which althougth we consider interaction between the magnetic charges which constitute the magnetic dipoles, we do not consider inter-dipolar interaction. This would be named a non-interacting system from the many-body Physics point of view. In this section we go to progress in the analysis and we study the system by means of an interacting dipole system with a many-body structure for the ground state and the corresponding low energy excitation states. Therefore, the results and the analysis have another complexity, and although, the results are coherent with those of section 2, the nature of the states presents novelties respect to that of the states of the section 2.

Within the magnetic structure scheme, we can define the following fermion operators ${{\rm{p}}}^{\dagger }|{\rm{0}}\rangle =|{\rm{p}}\rangle$ and ${{\rm{a}}}^{\dagger }|0\rangle =|{\rm{a}}\rangle$. Then, the many-body state of the gas constituted of confined magnetic dipoles can be defined in similar way to different many-body states in which pairs of particle-antiparticle are associated in a condensate such as the exciton bubble^17–19^. This is carried out by means of the following wave function:

9 $$|{{\rm{\Phi }}}_{0}\rangle =\prod _{{\rm{i}}}^{{\rm{N}}}\,({{\rm{\alpha }}}_{{\rm{i}}}+{{\rm{\beta }}}_{{\rm{i}}}{{\rm{p}}}_{{\rm{i}}}^{\dagger }{{\rm{a}}}_{{\rm{i}}}^{\dagger })|\mathrm{0,}\,0\rangle $$

where ${{\rm{\beta }}}_{{\rm{i}}}^{2}$ is the probability of obtaining the pair of magnetic charges, ±g_i_ in two contiguous tetrahedrons generated by a spin-flip in a vertex and, in addition, ${{\rm{\alpha }}}_{{\rm{i}}}^{2}+{{\rm{\beta }}}_{{\rm{i}}}^{2}=1$. The difference between this global wavefunctions from that of standard BCS- theory^21–24^ is that while the wave- function of confined dipoles is static, those of the BCS corresponds to associations or coupling between two particles which travel inside the crystal with k and -k linear momentum. In addition, the Hamiltonian which governs the system is in this case should exclude the posibility of several charges of the same sign within any tetrahedon since the repulsive interaction whitin the small space of a tetrahedron is very large, therefore, this Hamiltonian can be defined from the following expression:

10 $${\rm{H}}=\sum _{{\rm{i}}}^{{\rm{N}}}{\rm{\varepsilon }}({{\rm{p}}}_{{\rm{i}}}^{\dagger }{{\rm{p}}}_{{\rm{i}}}+{{\rm{a}}}_{{\rm{i}}}^{\dagger }{{\rm{a}}}_{{\rm{i}}})+\sum _{{\rm{i}}}^{{\rm{N}}}\,\sum _{{\rm{j}}}^{{\rm{N}}}\,{{\rm{V}}}_{{\rm{ij}}}{{\rm{p}}}_{{\rm{i}}}^{\dagger }{{\rm{a}}}_{{\rm{i}}}^{\dagger }{{\rm{a}}}_{{\rm{j}}}{{\rm{p}}}_{{\rm{j}}}+{\rm{U}}\sum _{{\rm{i}}}{{\rm{n}}}_{{\rm{i}}}^{{\rm{p}}}{{\rm{n}}^{\prime} }_{{\rm{i}}}^{{\rm{p}}}+{\rm{U}}\sum _{{\rm{j}}}\,{{\rm{n}}}_{{\rm{j}}}^{{\rm{a}}}{{\rm{n}}^{\prime} }_{{\rm{j}}}^{{\rm{a}}}$$

where V_ij_ < 0 corresponds to the attractive pole-antipole interaction. Without losing generality and for the sake of simplicity, we consider the isotropic version which can be simplified by V_ij_ = −|V_0_|, and this version will be utilizted with a many-body state of Eq. (9) based on N-bosons where each one-body state is a fermion pair. The two latter terms of the Hamiltonian can be interpreted as Hubbard-like terms which hinder two magnetic charges of the same sign could be within the same tetrahedron. The great repulsive energy U forbids the existence of two charges of equal sign within a same tetrahedron. In these two Hubbard-like hamiltonian terms, n_i_ and ${{\rm{n}}^{\prime} }_{{\rm{i}}}$ are two magnetic charges of the same sign arising from two spin-flips within the same tetrahedron.

These two Hubbard-like terms imply that only one of the seven vertexes of each pair of contiguous tetrahedrons can initially have a spin flip into the many-body excited condensate state of above wavefunctions. The average energy of the isotropic version of Hamiltonian (10) considering the many-body wave-function (9) and operating in similar way to that of the BCS theory [as in refs^21–23^ is:

11 $${\rm{E}}=\sum _{{\rm{i}}}^{{\rm{N}}}2{{\rm{\varepsilon }}}_{{\rm{i}}}{{\rm{\beta }}}_{{\rm{i}}}^{2}-|{{\rm{V}}}_{0}|\sum _{{\rm{i}}}^{{\rm{N}}}\sum _{{\rm{j}}}^{{\rm{N}}}{{\rm{\alpha }}}_{{\rm{i}}}{{\rm{\beta }}}_{{\rm{i}}}{{\rm{\alpha }}}_{{\rm{j}}}{{\rm{\beta }}}_{{\rm{j}}}=2{{\rm{N}}{\rm{\varepsilon }}{\rm{\beta }}}^{2}-\frac{{{\rm{\Delta }}}_{0}^{2}}{|{{\rm{V}}}_{0}|}$$

where ${{\rm{\Delta }}}_{0}=|{{\rm{V}}}_{0}|{\sum }_{{\rm{i}}}^{{\rm{N}}}{{\rm{\alpha }}}_{{\rm{i}}}{{\rm{\beta }}}_{{\rm{i}}}$, the energy ε_i_ is the potential energy $|{{\rm{g}}}_{{\rm{i}}}|{\rm{\varphi }}({\overrightarrow{{\rm{r}}}}_{{\rm{i}}})$ due to magnetic potential ${\rm{\varphi }}({\overrightarrow{{\rm{r}}}}_{{\rm{i}}})$ generated by the other charges of the system. This energy ε_i_ is deduced in the supplementary material given along with this paper. When the number of pairs n_i_ is close to the maximum value, the spin-flips can run over the crystal when the temperature increases, the lengths of the Dirac strings augment along with the probability of evaporating charges of the condensate and as a consequence, a new many-body excited state of higher temperature starts to be formed.

## Transition From the Dipolar State (9) Towards a Monopolar Plasma State

The form that has the experimental evolution with temperature of the specific heat of the lanthanide compounds crystallizing as spin-ices is characterized by the existence of a very narrow and intense peak between 0.05 and 0.2 K^25–29^. This seems an unequivocal empirical result and leads us to the idea that the formation of pole-antipole pairs can reach its maximum value at these very low temperature. This maximum has to have a limit value taking into account that the Hamiltonian of the Eq. (3) implies that any tetrahedron can only be associated with a single contiguous tetrahedron of the four possible in which it shares a spin-flip.

Exceeding this temperature of about 0.2 K, the length of the dipoles increases due to the evolution of the spin-flips such as we have mentioned in the previous paragraphs. Then, the number of free magnetic charges can progressively increase taking into account that it is equally probable the creation of positive and negative charges. This tends to create a gas of positive and negative charges in such a way that the total charge will remain zero. As a result, a state in the form of a magnetic plasma is progressively generated^30^, which can initially share space with the gas of pole-antipole pairs. Having in mind this idea, we go to consider the analytical principle of the fermion gas formation by means of the creation of fermionic particles from the $|{{\rm{\Phi }}}_{0}\rangle$-state of Eq. (9). This idea requires to establish analytically the evaporated charges) of the condensate^21,31^ whose evaporation process can be defined by means of the following creation operators applied to the $|{{\rm{\Phi }}}_{0}\rangle$- state:

12 $$\begin{array}{rcl}{{\rm{u}}}^{\dagger } & = & {\rm{\alpha }}\,{{\rm{p}}}^{\dagger }-{\rm{\beta }}\,{\rm{a}}\\ {{\rm{v}}}^{\dagger } & = & {\rm{\alpha }}\,{{\rm{a}}}^{\dagger }+{\rm{\beta }}\,{\rm{p}}\end{array}$$

definitions determine the corresponding annihilation operators, in such a way that we have the following operations over the ground state which is the formalization of an evaporated magnetic charge

13 $${{\rm{u}}}_{{\rm{j}}}^{\dagger }|{{\rm{\Phi }}}_{0}\rangle =|{{\rm{p}}}_{{\rm{j}}}\rangle \prod _{{\rm{i}}\ne {\rm{j}}}^{{\rm{N}}}({{\rm{\alpha }}}_{{\rm{i}}}+{{\rm{\beta }}}_{{\rm{i}}}{{\rm{p}}}_{{\rm{i}}}^{\dagger }{{\rm{a}}}_{{\rm{i}}}^{\dagger })|\mathrm{0,}\,0\rangle ,$$

simplifying the notation, we can write ${{\rm{u}}}^{\dagger }|{{\rm{\Phi }}}_{0}\rangle =|{\rm{p}}\rangle$, ${{\rm{v}}}^{\dagger }|{{\rm{\Phi }}}_{0}\rangle =|{\rm{a}}\rangle$,${\rm{u}}|{{\rm{\Phi }}}_{0}\rangle =0$ and ${\rm{v}}|{{\rm{\Phi }}}_{0}\rangle =0$, where $|{\rm{p}}\rangle (|{\rm{a}}\rangle )$ is an excited state respect to the BC ground state $|{{\rm{\Phi }}}_{0}\rangle$ corresponding to the creation of a positive (negative) free magnetic charge and which are the magnetic charges evaporated from the condensate. The many-body state with M/2 free positive magnetic charges, a M/2 free negative ones and M′ confined dipoles till inside the condensate is:

14 $$|{\rm{\Phi }}\rangle =\prod _{{\rm{i}}}^{{\rm{M}}\mathrm{/2}}{{\rm{u}}}_{{\rm{i}}}^{\dagger }\prod _{{\rm{j}}}^{{\rm{M}}\mathrm{/2}}{{\rm{v}}}_{{\rm{i}}}^{\dagger }|{{\rm{\Phi }}}_{0}\rangle =\prod _{{\rm{i}}}^{{\rm{M}}\mathrm{/2}}\,{|{\rm{p}}\rangle }_{{\rm{i}}}\prod _{{\rm{j}}}^{{\rm{M}}\mathrm{/2}}\,|{{\rm{a}}}_{{\rm{j}}}\rangle \prod _{{\rm{l}}}^{{\rm{M}}^{\prime} }\,({{\rm{\alpha }}}_{{\rm{l}}}+{{\rm{\beta }}}_{{\rm{l}}}{{\rm{p}}}_{{\rm{l}}}^{\dagger }{{\rm{a}}}_{{\rm{l}}}^{\dagger })|\mathrm{0,}\,0\rangle $$

where M + M′ =N′, N′ being the number of vertexes of the tetrahedons. This above wave-function state (14) is representative of system with two fluids. The average value of hamiltonian Eq. (10) yields an energy whose expression can be easily determined via the definitions of the actions of creation and destruction operators defined, this expression is the following:

15 $${\rm{E}}\approx 2\sum _{{\rm{i}}}^{{\rm{M}}^{\prime} }[{{\rm{\varepsilon }}}_{{\rm{i}}}{{\rm{\beta }}}_{{\rm{i}}}^{2}-\frac{{{\rm{\Delta }}}_{0}^{2}}{2{\rm{M}}^{\prime} |{{\rm{V}}}_{0}|}]+\sum _{{\rm{i}}}^{{\rm{M}}}[{{\rm{\varepsilon }}}_{{\rm{i}}}\mathrm{(1}-2{{\rm{\beta }}}_{{\rm{i}}}^{2})+\frac{{{\rm{\Delta }}}_{0}^{2}}{\sqrt{{\langle {\rm{\varepsilon }}\rangle }^{2}+{{\rm{\Delta }}}_{0}^{2}}}]$$

where the one body energy ε_i_ corresponding to free magnetic charge of Eq. (2), excluding the self energy per magnetic charge since this term is not dependent on temperature. In order to describe the temperature evolution of the extensive state functions and their corresponding first and second temperature derivatives, in this scenario, we have considered the statistical thermodynamic theory in order to determine the specific heat and entropy starting from the thermodynamic potential or Helmholtz free energy F. We determine the thermodynamic Hemholtz potential for a mixed fluid forming two channels one of them as a boson gas and another one constituted of fermions^30,32^ described by the many-body functions of Eq. (14):

16 $${\rm{F}}=-\,\frac{1}{{\rm{\beta }}}\{2\sum _{{\rm{j}}}\,\mathrm{ln}[1+\exp (-{\rm{\beta }}({{\rm{e}}^{\prime} }_{{\rm{j}}}-{\rm{\mu }}^{\prime} ))]-\sum _{{\rm{j}}}\,\mathrm{ln}[1-\exp (-{\rm{\beta }}({{\rm{e}}}_{{\rm{j}}}-{\rm{\mu }}))]\}$$

where, ${{\rm{e}}^{\prime} }_{{\rm{j}}}$ and e_j_ are the one-body energies of the fermions and bosons, respectively i.e. the monopoles and dipoles respectively corresponding to the mixed fluids of many-body wavefunctions of Eq. (14), whose expressions are given by:

17 $${{\rm{e}}}_{{\rm{j}}}=2({{\rm{K}}}_{{\rm{\beta }}}{\rm{Tln}}(\frac{{{\rm{n}}}_{0}}{{{\rm{n}}}_{{\rm{j}}}})+{\rm{T}}\frac{|{\rm{g}}|{{\rm{\varphi }}}_{0}}{{{\rm{T}}}_{{\rm{V}}}}){{\rm{n}}}_{{\rm{j}}}+{{\rm{\Sigma }}}_{0}-\frac{{{\rm{\Delta }}}^{2}}{|{{\rm{NV}}}_{0}|}$$

18 $${{\rm{e}}^{\prime} }_{{\rm{j}}}=({{\rm{K}}}_{{\rm{\beta }}}{\rm{Tln}}(\frac{{{\rm{n}}}_{0}}{{{\rm{n}}}_{{\rm{j}}}})+{\rm{T}}\frac{|{\rm{g}}|{{\rm{\varphi }}}_{0}}{{{\rm{T}}}_{{\rm{V}}}})(1-2{{\rm{n}}}_{{\rm{j}}})+{{\rm{\Sigma }}}_{0}+\frac{{{\rm{\Delta }}}^{2}}{{({\langle {{\rm{e}}}_{{\rm{j}}}\rangle }^{2}+{{\rm{\Delta }}}^{2})}^{1/2}}$$

then, the Helmholtz free energy per channel can be given by the following expressions (see the supplementary material of this paper):

19 $${{\rm{F}}}_{{\rm{BEC}}}={\rm{N}}^{\prime} \frac{{{\rm{K}}}_{{\rm{B}}}{\rm{T}}}{2{{\rm{n}}}_{0}}{\int }_{0}^{2{{\rm{n}}}_{0}}\,\mathrm{ln}|1-{(\frac{{\rm{t}}}{2{{\rm{n}}}_{0}})}^{t}{{\rm{e}}}^{-{\rm{at}}}{{\rm{e}}}^{-{\rm{b}}{\rm{\beta }}}|{\rm{dt}}$$

20 $${{\rm{F}}}_{{\rm{Plasma}}}=-\,{\rm{N}}^{\prime} \frac{{{\rm{K}}}_{{\rm{B}}}{\rm{T}}}{2{\rm{m}}}{\int }_{1-2{\rm{m}}}^{1}\,\mathrm{ln}[1+{(\frac{1-{\rm{t}}}{2{{\rm{n}}}_{0}})}^{{\rm{t}}}{{\rm{e}}}^{-{\rm{at}}}{{\rm{e}}}^{-{\rm{b}}^{\prime} {\rm{\beta }}}]{\rm{dt}}$$

where, ${\rm{a}}=\frac{|{\rm{g}}{{\rm{\varphi }}}_{0}|}{{{\rm{K}}}_{{\rm{B}}}{{\rm{T}}}_{{\rm{V}}}}$, ${\rm{b}}={{\rm{\Sigma }}}_{0}-\frac{{{\rm{\Delta }}}_{0}^{2}}{{\rm{N}}|{{\rm{V}}}_{0}|}-{\rm{\mu }}$, ${\rm{b}}^{\prime} ={{\rm{\Sigma }}}_{0}+\frac{{{\rm{\Delta }}}_{0}^{2}}{\sqrt{{\langle {\rm{\varepsilon }}\rangle }^{2}+{{\rm{\Delta }}}_{0}^{2}}}-{\rm{\mu }}$, n_0_ is the probability that a magnetic dipole is created in each pair tetrahedron in the bosonic condensate state, m is the probability of appearing a free magnetic charge coming from a confined dipole of the BC state. Having in mind these definitions, the entropy in the boson channel per each vertex in which a spin-flip is possible, taking into account that all vertexes have the same probability for undergoing a spin-flip due to the temperature variation, is given by:

21 $${{\rm{s}}}_{{\rm{BEC}}}({\rm{T}})=-\,\frac{1}{{\rm{N}}}\frac{{{\rm{dF}}}_{{\rm{BEC}}}}{{\rm{dT}}}$$

and the entropy for each dipole of the bosonic phase when it is decaying and is becoming the fermionic phase of two free charges can be given by the following expression:

22 $${{\rm{s}}}_{{\rm{Plasma}}}({\rm{T}})=-\,\frac{2}{{\rm{N}}^{\prime} }\frac{{{\rm{dF}}}_{{\rm{Plasma}}}}{{\rm{dT}}}$$

The specific heat in the boson channel corresponding to the BC global state per effective dipolar component is:

23 $${{\rm{c}}}_{{\rm{BEC}}}({\rm{T}})=-\,\frac{1}{{\rm{N}}^{\prime} }{\rm{T}}\frac{{{\rm{d}}}^{2}{{\rm{F}}}_{{\rm{BEC}}}}{{{\rm{dT}}}^{2}}$$

On the other hand, the specific heat of the fermionic channel of the magnetic plasma state per each free magnetic charge is given by:

24 $${{\rm{c}}}_{{\rm{Plasma}}}({\rm{T}})=-\,\frac{1}{{\rm{N}}^{\prime} }{\rm{T}}\frac{{{\rm{d}}}^{2}{{\rm{F}}}_{{\rm{Plasma}}}}{{{\rm{dT}}}^{2}}$$

The development of calculation of Eqs (19)–(24) is given in the supplementary material adjoint to this paper.

An important characteristic of the bosonic channel, not present in the fermionic one, is according to Eq. (19), the possibility of presenting divergences both in the thermodynamic potential and in its derivatives regarding the temperature. These divergences define first-order transitions due to divergences in the thermodynamic potential, internal energy and the specific heat^25–29^. This is not a minor issue neither from the theoretical point of view nor much less from its empirical consequences. Indeed, in the Eq. (19) of the bosonic channel, the possibility of infinite values appears at a certain temperature when the following equality is fulfilled in the integrals:

25 $$1={(\frac{{\rm{t}}}{2{{\rm{n}}}_{0}})}^{{\rm{t}}}{{\rm{e}}}^{-{\rm{at}}}{{\rm{e}}}^{-{\rm{b}}{\rm{\beta }}}={(\frac{{\rm{n}}}{{{\rm{n}}}_{0}})}^{2{\rm{n}}}{{\rm{e}}}^{-2{\rm{an}}}{{\rm{e}}}^{-{\rm{b}}{\rm{\beta }}}$$

When this equality is satisfied for a value of temperature, the internal energy and the specific heat are representative of a first-kind phase transition. On the contrary, in the fermionic channel this possibility does not exist since in Eq. (20), any divergence is not possible. Therefore, the value of the interaction energies among magnetic charges plus their energy of formation, defined as self-energy Σ_0_ in the dumbbell model^1,2^, are causes that collaborate in the generation of a phase transition of either first or second order at T_V_-temperature in a similar but different way to that of section 1. In other words, this energy b and the parameters a and n_0_ determine if in the specific heat there is an infinite or a simple cusp or possible discontinuity in the derivatives with respect to the temperature of the thermodynamic potentials.

We want to emphasize that the expressions of specific heat given in Eqs (23) and (24) is specially sensitive to the variations of the energies, b, and b′ as well as the parameters a, n_0_ and m such as we have said in the last paragraphs of the previous section. This deserve to coment about the possible intervals in which the values of these energies and parameters are in actual cases.

The dimensionless parameter,a, establishes the relationships between the “magnetronic” potential energy of the magnetic system interacting with the magnetic charges and the thermal energy at temperature corresponding to first maximum of the specific heat. This temperature at which the maximum appears is one in which the lowest energy state is dominant. This state, as above mentioned, may be a Bose-Einstein condensate in similar way to that proven in section 2 and we analyze below again^33–38^. The temperature evolution leads to a system towards another low-energy excited state which is similar to a plasma state where the currents of magnetic monopoles can be defined as ${{\rm{J}}}_{{\rm{m}}}=\partial {\rm{M}}/\partial {\rm{t}}$ ^30,39^. The first of these states being the culminating point in the specific heat corresponding to the narrowest peak. The possible values of this a- parameter should be in the interval between the unity and the relation T_V_/T_c_ whose value is around 0.1, since the two energy amounts of the corresponding fraction ${\rm{a}}=|{\rm{g}}|{{\rm{\varphi }}}_{0}/{{\rm{K}}}_{{\rm{B}}}{{\rm{T}}}_{{\rm{V}}}$ is referred to energies typical of the corresponding states (T_C_ being temperature of the monopolar maximum in c_V_).

The parameters n_0_ and m are the probabilities for the presence of the confined magnetic dipoles at T_V_ which is generated via the corresponding spin-flip and the probability for the breaking of each pair in the two magnetic charges at T_C_, respectively. The values of the n_0_ and m can be obtained from indirect experimental results arising from the magnetic conductivity (see refs^7,8^ and references therein) they may be between 0.25 and 0.75 in the case without first order phase transition. The energies b and b′ are those energies which do not depend on the increasing of the probability of appearance of the magnetic charges due to the temperature evolution. They are consequence of magnetostatic energies which comes from the gg′ interactions^33^. And last, but not least, μ is the chemical potential defined as ${\rm{\mu }}=\partial {\rm{F}}/\partial {\rm{N}}$. The values of this chemical potential is more difficult to obtain by means of experimental data in the monopolar chanel, although in the case of BEC state, this chemical potential is zero, such as it occurs in the case of section 2, when z = 1.

## Comments on the Results

These confined dipoles have complete similarity with microimagnets, or better said nanomagnets of dimensions of the height of each tetrahedron. When the temperature continues increasing the spin-flips of the magnetic moments are propagated along the magnetic structure. Then the length of the namomagnets increases until true free charges coexist (monopoles and antimonopoles) with confined dipoles. The confined dipoles have a structural resemblance to the mesons^16^ with the difference that the quarks and anti-quarks that form the hadronic structure can not be released from each other, while the poles and anti-poles at spin-ices can be converted into effective free magnetic charges either under increasing temperatures or the presence of external magnetic fields. Then, there is coexistence between two quantum fluids, one of them corresponding to confined dipoles and another one to free monopoles. That is why we configure the model of two fluids in which the different form of Helmholtz free energy^32^ corresponding to the bosonic fluid (of confined pole-antipole pairs) competes with the fermion gas constituted of positive and negative free charges.

In addition, the charges of different sign are quasi-particles with +|g| and −|g| which have the same energy. This energy equality is because their presence is simultaneously generated and whose energy is that necessary to produce the spin-flip. There exists the experimental doubt about whether the lowest-temperature structure in the specific heat of the spin-ices corresponds to either a first or second order phase transition, and even if it is due to a simple structure due to an sudden increase of confined dipoles in a low-energy excited global state.This doubt is logical since its existence occurs at very low temperature, between 0.05 and 0.2 K^25–29^. When Eq. (25) is satisfied, the specific heat suffers a divergence due the existence of an essential infinite for an intermediate value in integrals which define it (see these integrals in the supplementary material adjoint this paper). Having in mind Eq. (25) and given the meaning of these variables, the condition for the existence of a first-order phase transition is:

26 $$\frac{{\rm{b}}}{{{\rm{KT}}}_{{\rm{V}}}}=2{\rm{n}}({\rm{lnn}}-{{\rm{lnn}}}_{0}-{\rm{a}})$$

where all constants of this equation have been defined and n is the variable of integration which defines a determined probability for the spin-flips to be generated in the corresponding state. Having in mind the meaning of parameters intervining in this Eq. (26), this can be transformed in the following condition for obtaining the Boson Einstein condensate:

27 $$2|{\rm{g}}|{\varphi }_{0}{{\rm{n}}}_{0}+{{\rm{\Sigma }}}_{0}-\frac{{{\rm{\Delta }}}_{0}^{2}}{{\rm{N}}|{{\rm{V}}}_{0}|}=0$$

This condition is perfectly coherent with the corresponding one given in the section 2, when we explain the condition of appearing the Bose Einstein condensation in Eq. (7) and Fig. 1, i.e. when we consider a many-body system of independent particles and the differences are due to the inter-dipole interaction of Hamiltonian (10) which is included in section 3 and whose contibution is represented by $\frac{{{\rm{\Delta }}}_{0}^{2}}{{\rm{N}}|{{\rm{V}}}_{0}|}$. Eq. (26) implies a divergence and essential discontinuity in the specific heat which implies that all possible confined dipoles are in the boson condensate and the energy of the individual bosons have a zero energy according to Eq. (17). Therefore, the conditions which a global bosonic state is a Bose-Einstein condensation can once again been satisfied when one consider the inter-dipole interaction.

**Figure 2:**
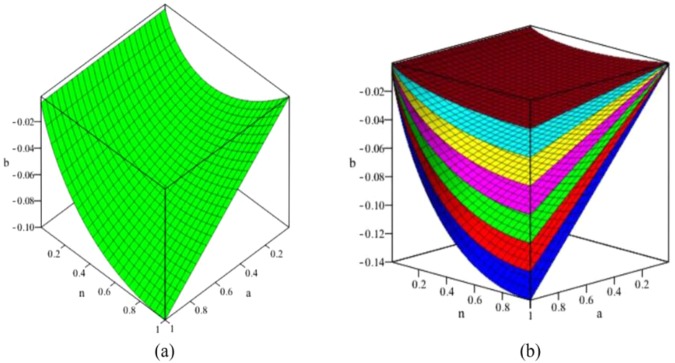
In (**a**) we give a surface corresponding to Eq. (26) for T_v_ = 0.1 K and n_0_ = 0.5. For each point in this surface defined for its coordinates: the a-parameter, the b-energy and the n- variable, there exist a divergence which implies the existence of a first-order phase transition, which is manifested in the specific heat for the given temperature T_v_ and a given probability of pair formation. n_0_. In (**b**), we given seven examples of seven surfaces corresponding to n_0_ = 0.1 (brown); n_0_ = 0.2 (cyan), n_0_ = 0.3 (yellow), n_0_ = 0.4 (magenta); n_0_ = 0.5 (green); n_0_ = 0.6 (red) and n_0_ = 0.7 (blue).

If we represent Eq. (26) in a parameter space, considering a given T_V_ whose value can be between 0.05 and 0.17 K. according to our results and a given value for the maximum n_0_, we can construct the parameter space with b as Z axis, a as Y axis and n the variable in the integrals as X axis. Then, Eq. (26) is represented in Fig. 2.

In Fig. (2), we give a surface in the 3-dimensional parameter space whose points in this surface describe the physical situations in which the specific heat has an essential infinite and therefore, it derives towards a first order phase transition. The values of the b-energy and the a-parameter can imply the existence of a n-value which constitutes a point in the parameter space located on the surface of Fig. 2a or on any other surface similar to those of Fig. 2b. These values being actual parameters in real spin-ice compounds. If under normal conditions it is not possible, when the system is subjected to hydrostatic pressures or in the presence of external magnetic fields, then the divergences in the specific heat can appear.

The low temperature (between 50 and 170 mK), at which these first-order thermodynamic transitions must appear, are the cause for which they have not been profusely detected, and although detected, the phase transition order is not explained with total certainty. However, in 2016 an experimental result^40^ detected, according to authors, structural modifications at temperatures below 1 K, with a first-order transition signature without the existence of changes in the crystalline structure. The other points of the cube of the parameter space of Fig. 2, the phase transition, if any, will be of second order. This Fig. 2 can be made for different T_V_ and n_0_ (see Fig. 2b) values but the shape being quantitatively different is qualitatively similar.

With the results that we give below, we do not intend to present them as novelties. Our objective is to weigh the possibilities of our analytical model and to use fundamental principles of double quantum fluids comparing our results with others made with Monte Carlo statistical models^25,26,41–43^ or other experimental methods^27,42,44^. In our opinion, the important thing in this document is in the ingredients of the model and the different analyzes that can be done with it. The results that we present are examples and we only try to put the methodology into practice. We believe that our model goes a little further in the knowledge of the nature of the global excited states of the system and the possibilities of obtaining more approximate results by modifying the parameters that govern the fundamental equations.

Therefore, henceforth, we will stick and limit to the points of the parameter space in which there are no divergences, consequently we will concentrate on the situations which are outside the surfaces of Fig. 2a,b. These cases seem to correspond to thermodynamic situations whose signature is close to a phase transition of the second order in which a large number of previous theoretical and experimental results give graphic representations of the specific heats that agree with our results^1,25,26,45^.

**Figure 3:**
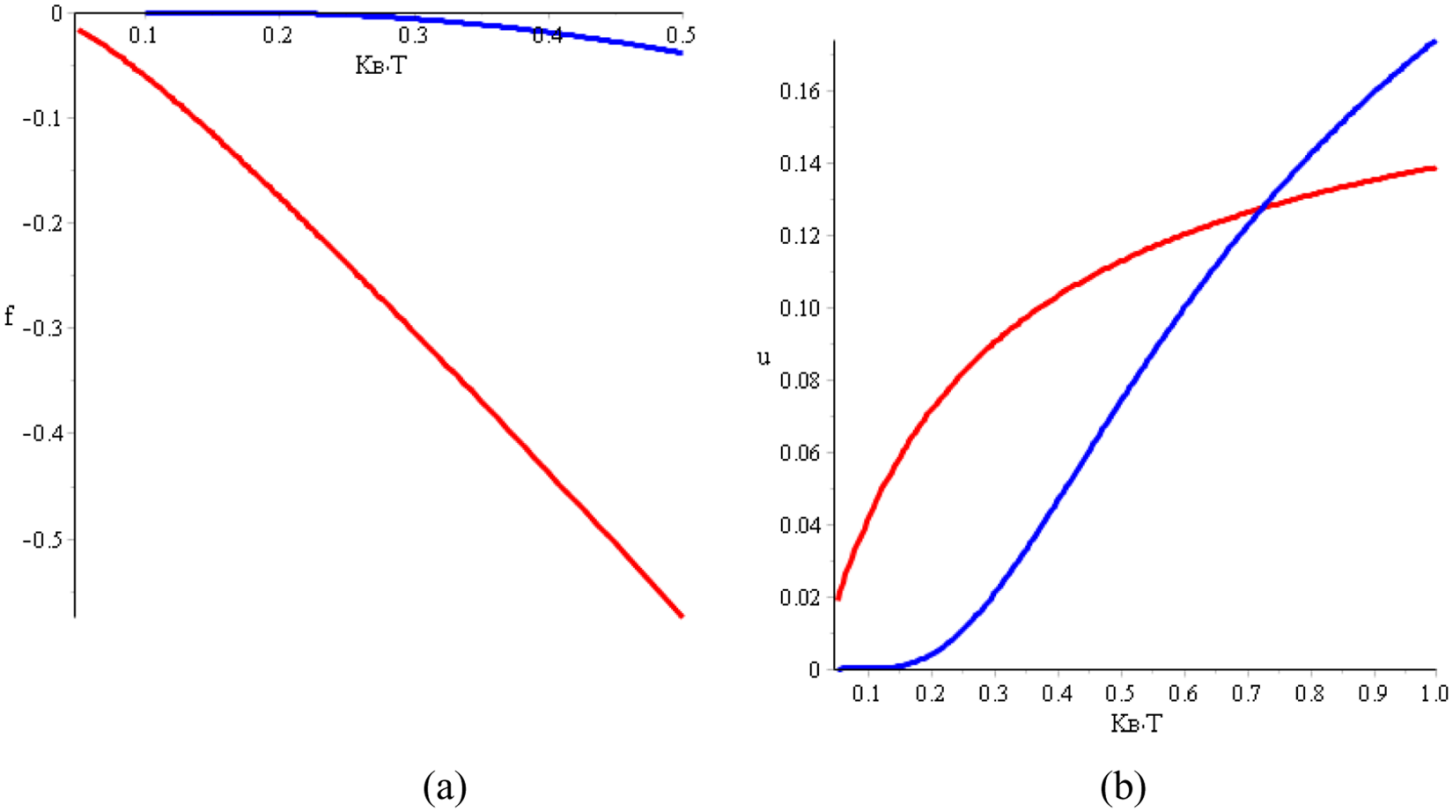
Evolution with temperature of the thermodynamical potential and internal energy per particle. In Fig. 3, with the red line, we represent the internal energy and the thermodynamical potential of the bosonic phase of confined magnetic dipoles as pairs of pole-antipole joined by the attractive interaction between both^30,43,47,48^. With the blue line we represent these two physical magnitudes per particle with magnetic charge in the free state. In Fig. 2a, we give the thermodynamic potential for the values of parameters a = 0.1, n_0_ = 0.5, m = 0.5 and energies b (b′) = 0.05 (1.00) in the X-axis units. The blue line corresponds to the thermal evolution of the fermion channel thermodynamic potential and the red line corresponds to he same physical magnitude of the boson channel. (**b**) corresponds to the thermal evolution of the internal energy with the same parameter and energies considered in (**a**). The red curve is the bosonic channel and the blue line corresponds to the fermionic channel.

The different evolution of the thermodynamic potential and internal energy (see Fig. 3) both at very low temperature is the reason for the existence of a possible discontinuity in the derivative of the Helmholtz free energy which will lead, if any, to these phase transitions of second order. The physical reason for the different evolution with the temperature of both free energies is typical and due to the different nature of the thermodynamic potentials in the bosonic case with respect to the fermionic one. This difference being the fundamental reason that generates the features of the free and internal energies of the two phases, which in turn induces the special evolution of the specific heat.

**Figure 4:**
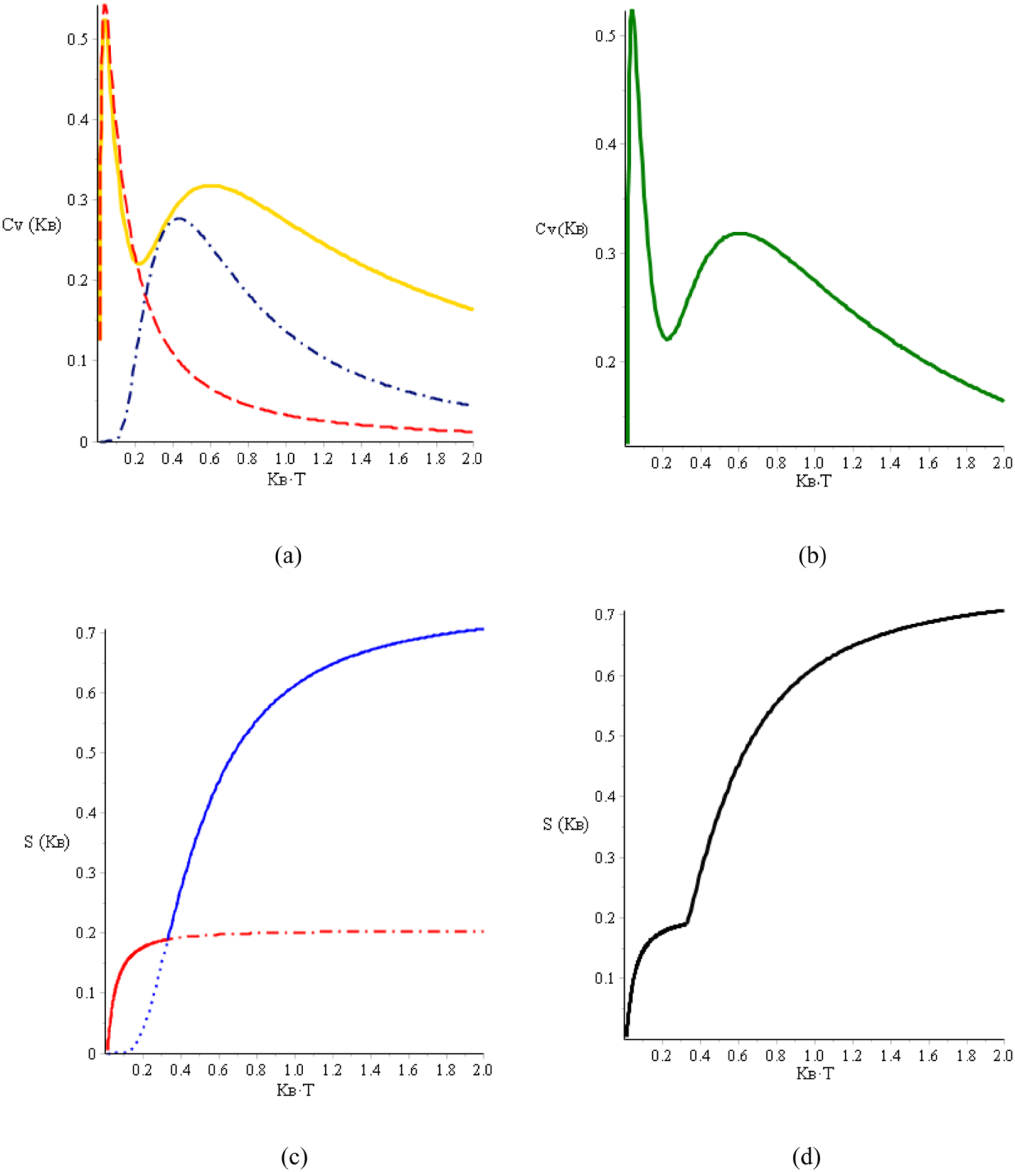
In (**a)**, we give the specific heat in the two first kelvins:the discontinuous red curve of narrow and intense peaks corresponds to specific heat c_BEC_ (T) of the boson channel. This presents a rapid increase of the pole-antipole pairs and the also fast breaking of those dipoles via the prolongation of the splitting among the two poles but still considered partially confined dipoles. The discontinuous blue curve of c_plasma_ (T) of the fermionic channel presents the second wider and less intense peak. The continuous yellow curve, we represent ${\rm{c}}({\rm{T}})=(1-\frac{{\rm{T}}}{{{\rm{T}}}_{{\rm{c}}}}){{\rm{c}}}_{{\rm{B}}}({\rm{T}})+2(\frac{{\rm{T}}}{{{\rm{T}}}_{{\rm{C}}}}){{\rm{c}}}_{{\rm{P}}}({\rm{T}})$. This is the complete specific heat in which we suppose with this calculation the idea of a progressive and continuous breaking of dipoles and formation the free magnetic charges. In (**b)**, we draw the curve of c_BEC_ (T) until it is cut with the curve c_plasma_ (T) which continue until 2 K. In (**c**), we represent the entropy per component; the blue curve is the corresponding entropy to the fermion channel and the red curve is that of the boson or confined dipole channels according to Eqs (23) and (24). The discontinuous curves are the prolongation of the entropy to different channels. In (**d**), we represent the entropy taking into consideration the change of Pauli symmetry within the the magnetic charge fluids. The first part of the curve up to 0.35 K corresponds to the bosonic contribution to the entropy and from this temperature value, the confined dipoles are progressively disappearing such as occurs in the specific heat, and the free magnetic charges emerge. The values of parameters defined in Eqs (19) and (20) which we have considered to obtain the results of this figure are the following: a = 0.1, n = 0.5, m = 0.7, b′(b) = 1.00(0.05) where these energies are given in the X- axis units.

In the ground state the number of confined dipoles in each pair of two contiguous tetrahedrons must be zero and therefore the specific heat at zero kelvins should be null, as it appears in our results as well as in other investigations^25,42,46^. On the contrary, in other experimental results^27,47^ and other obtained by statistical procedures as in refs^26,44^ this logic characteristic is not reflected in their results. Perhaps the low temperatures in the experimental results and the problems of establishing the specific heat when the growth of the internal energy is so intense at temperatures lower than 50 mK prevents obtaining results adjusted to the expected. The main and more significative difference in both the internal energy and the Helmholtz function between the two curves corresponding to the bosonic and fermionic states is in the temperature interval between 0 and 0.2 K. In this narrow interval, the two energies corresponding to the fermionic phase are practically null, while that energy of the bosonic phase is fast increasing. This difference is the cause for the existence of the intense peaks in the specific heat of Fig. 4a,b and the possible discontinuity in the derivative versus temperature of the Fig. 4b–d.

In this narrow interval of temperature between 0 and 70 mK the curve of specific heat is based on the fast increasing of components of bosonic states. This abrupt growth reaches the peak, from which the number of dipole components abruptly decreases, beginning the creation of Dirac’s strings and with the increasing temperatures, the free magnetic charges emerge. When the specific heat curve reaches the minimum this creation accelerates obtaining an evident growth of free charges but with less intensity than the most intense and narrow maximum. This image is the main characteristic of the spin-ices’ specific heat^25–27,42,44,48^. It is compatible with the description of a transition of the bosonic condensation to the gas of free magnetic charges by means of the evaporation of the magnetic charges from the dipole condensate at this low temperatures. The change of dominant state can be clearly seen at around 0.20 K, the narrow peak is centered at around 70 mK and the second wide maximum is at 0.55 K, this latter datum being in concordance with ref.^1^. These results being in qualitative concordance with those of experimental data of the literature (for instance, see refs^25–27,42–44^).

The figures of entropy deserve to be commented and related to those of specific heat. This evolution of entropy under temperature increasing giving in Fig. 4c is compatible with that of the specific heat. In this figure, the red line refers to the growth of entropy due to the increase of the creation of magnetic pole-antipole pairs and practically null presence of free magnetic monopoles. When this entropy of the bosonic channel no longer increases, arrives at a constant that is reached around T = 0.35 k in Fig. 4c and its value is around 0.20, [0.5In(1.5)]K_B_. This value coincides with the residual Pauli entropy which is assigned in water ice to the degeneration its the ground state. From this value, the bosonic phase disappears and the fermionic channel of free charges is responsible of the energy increase. Then, entropy due to the fermionic gas grows until it reaches the value of the saturation entropy per free magnetic charge.

Therefore the cut between the two entropy curves is done with a discontinuity in its derivative respect to the temperature. It may be consistent with the existence of a phase transition of states with different symmetry with respect to the spin of the individual components such as it is shown in ref.^25^. Similar discontinuity appears in Fig. 4a,b when one joints the specific heat curves corresponding to the bosonic part (discontinuous red line) and the corresponding one of the fermionic channel (discontinuous blue line). However, the discontinuity on the derivative with temperature in both specific heat and entropy is very difficult to be experimentally observed given the extremely low temperatures in which they should be obtained. The coherence between two results of Fig. 4a,c as well as the 4b and 4d may have a certain importance since it gives support to the assumed hypotheses. This cut, in our results which occurs at approximately the same temperature (0.35 K similar to that of Fig. 4c,d), in a similar qualitative way as that of ref.^25^.

The yellow solid line in Fig. 4a is carried out in the sense that the change of spin symmetry between fluids may be continuous and not abrupt. In other words when one considers the conversion from the pairs in the creation of free charges in a continuous and progressive way, then we can represent a specific heat by means of a continuous mixing between the boson and fermion channels, and this can be carried out by means of the expression:

$${\rm{c}}({\rm{T}})=[1-{(\frac{{\rm{T}}}{{{\rm{T}}}_{{\rm{C}}}})}^{{\rm{x}}}]{{\rm{c}}}_{{\rm{BEC}}}({\rm{T}})+2{(\frac{{\rm{T}}}{{{\rm{T}}}_{{\rm{C}}}})}^{{\rm{x}}}{{\rm{c}}}_{{\rm{Plasma}}}({\rm{T}}\mathrm{).}$$

The results of yellow curve in Fig. 4a are realized using, for the sake of simplicity for x = 1. This idea of the mixing to establish a specific heat c(T) based on the continuous sum of the two contributions has a double motivation. A first physical reason is to consider the alternative idea that the change of spin symmetry from the boson channel to a fermion gas might occur in a continuous and progressive way; and a second reason is in to obtain an image for the specific heat more similar to that of experimental curves^26,27,42–44,47,48^.

## Some Agreements and Discrepancies with Previous Results

According to the classical theory of Thermodynamics, a n-order phase transition requires a discontinuity in a n-derivative of the Helmholtz thermodynamic potential with respect to temperature when all minor orders of derivatives are continuous. Therefore, a first-order phase transition requires a jump in entropy, and a second-order transition will be defined when the jump is in the derivative of entropy or in specific heat. However, the characteristic in the specific heat that is repeated in much of the analysis of these two thermodynamic variables is the presence of two structures in the form of two peaks, one narrow, intense between 0.05 and 0.2 K and another wider one whose maximum is separated from the first between 0.4 and 0.8 K. Only in two curves which correspond to two references between ten given in Fig. 3 of the work of Pomaransky *et al*.^27^ and in another one in McClarty *et al*.’s work^26^ seem to be the results of specific heat compatible with a phase transition of the first order Other results of Kato *et al*.’s work^25^ clearly shows a discontinuity in the derivative with respect to temperature of entropy which is compatible with a transition of the second order. However, there is some calculation^42^ whose results of C_V_ qualitatively agree with those of Fig. 4 and which their authors afirm the compatibility of their data with the existence of a transition of first order presenting results with the two structures in the specific heat although with continuous curves. Therefore, in Fig. 4 we give a result that always reflects the most repeated and incontrovertible fact as is the existence of the two structures in the specific heat and we give in section 1 and 4 two criteria for the existence of a first order phase transition.

**Figure 5:**
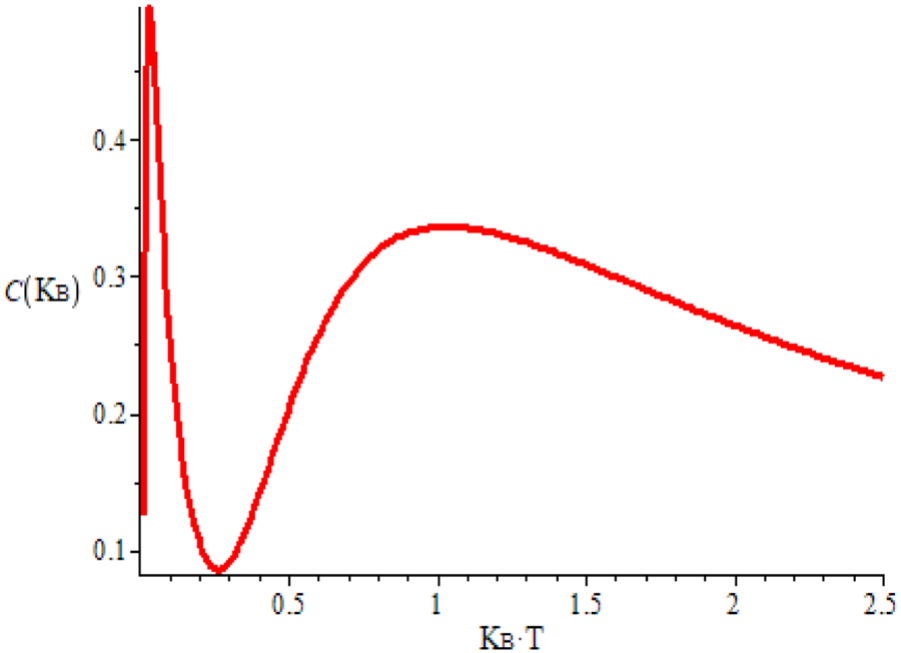
Specific heat in the continuous model (yellow curve of Fig. 4) but considering b′ = 1.5 in Eq. (20) instead of b′ = 1 of Fig. 4, leaving identical the remaining input data. In this Fig. 5, the specific heat due to the dipole channel becomes zero before the monopolar channel begins to grow. This fact can be interpreted considering that the specific heat of the dipole channel decreases when the dipoles are converted into Dirac’s stings. When the free charges begin to dominate is when the specific heat returns to grow. We believe that this result would be compatible with a second-order phase transition.

On the other hand, we look from the point of view of the difference in the evolution of Cv and S (in Fig. 4a,b) with the temperature in the dipolar phase with respect to those variables in the monopolar, a compatibility with a second order transition can be possible in the transition from the dipolar to the monopolar phases. This second transition disappears when we consider the progressive transition from the bosonic to the fermionic regime (see the yellow curve in Fig. 4 and the results of Fig. 5). However, what seems more important, in our opinion, is the qualitative form of evolution in the low energies of spin-ice system states. These excited states imply the two peaks that appear in the specific heat in practically all the works carried out since 2012 and we consider that, maybe, the quantitative values seem to be less important. Effectively, the curves of Cv from the Fig. 4 differ from those of the references given in Fig. 3 of the work of Pomaransky. This difference is due to have carried out these calculations with energy b′ = 1.0. However, Cv in Fig. 5 is realized with b′ = 1.5, leaving all the other parameters equal and as a consecuence, we obtain a result with much smaller differences from the cited references.

## Conclusions

Our research can be included within the genuine dumbbell model that is defined by the following logical segment: (i) in the first step, in vertices of tetrahedron pairs, spin-flips arise, which it implies the creation of confined magnetic dipoles under slight increases in temperature; (ii) in the second place Dirac’s strings are developed and in this stage, the second derivative of the thermodynamic potential increases and therefore the specific heat decreases; (iii) in the third step, free magnetic charges progressively appear and then, the specific heat again increases up to a new maximum. In this pattern, the first step is the concentration of bosons as the lowest energy excitation state, in the decreasing zone of specific heat corresponds to proliferation of the Dirac’s strings and in the second wide peak corresponds to the plasma state.

When a set of bosons whose number, N_0_, fulfill the condition $\mathop{\mathrm{Lim}}\limits_{{\rm{N}}\to \infty }\frac{{{\rm{N}}}_{{\rm{o}}}}{{\rm{N}}} > 0$, N being the maximum number of components of the set, this constitute a Bose-Einstein condensate if and only if these bosons occupy the lowest possible energy state^24^. We have deduced the conditions for which this concentration of bosons can become a Bose-Einstein condensate that is a macroscopic quantum state in which practically all the dipoles are in the ground state of the energy spectrum of a single body. These conditions for this conversion of states from BS to BEC are justified both in cases whose individual bosons are considered independent particles without interaction between them and when a Hamiltonian (10) is considered which includes inter-dipolar interaction. The conditions in both cases are different but compatible. In addition, in the latter case, a wave function that is inspired by the BCS theory is proposed^46^. By further increasing temperatures, the confined dipoles break progressively and then the free magnetic charges evaporate from BS or BEC and form a Coulomb gas of free charges of different signs whose structure is that of a cold magnetic plasma. The process of evaporation is formalized by means of a many-body state whose wave function^13^ is appropriate for a true two-fluid system. Then, we justify the formulation of the corresponding thermodynamic potential that contains the bosonic and fermionic channels and we form this free energy of Helmholtz and we determine the evolution with the temperature of the specific heat, the entropy, the internal energy that are reasonably in agreement with other previous calculations and experimental data.

We obtain the existence of a phase transition at around 70 mK determined in our results of Figs 4 and 5. This fact being perfectly qualitatively coherent with the experimental results^25–27^. This phase transition may be either a first order or a second order. We have given clear criteria for the discrimination between the possible kinds although, unfortunately, there is not still a complete experimental consensus about this order due to the extremely low temperatures at which the phase transition occurs. In any case, it is clear that at T = 0 K, the specific heat should be null and at temperatures less than 0.2 K there is a huge increasing of this thermodynamic variable such as it is given in our results, this fact in concordance with previous data^26,27,42,47^.

## Electronic supplementary material

Supplementary Material
