# Supplementary material for: Two fluid model in low energy excited states within spin-ice systems
Source: Sci Rep. 2018 Nov 2;8:16303. doi: 10.1038/s41598-018-34529-x (PMC6215019; doi:10.1038/s41598-018-34529-x)
Supplement: Supplementary file 1 — Supplementary Material [file 41598_2018_34529_MOESM1_ESM.docx]

**Supplementary material of Two fluid model in low energy excited states within spin-ice systems.**

**F.I. López-Bara, and F. López-Aguilar**

**Electromagnetism Group, Department of Physics, Autonomous University of Barcelona, Campus de Bellaterra, Bellaterra, Cerdanyola del Vallés, E-08193 Barcelona, Spain**

In this Supplementary paper, we give the derivations of some expressions and Equations corresponding to the paper: Two fluid model in low energy excited states within spin-ice systems. We explain the derivations of magnetic potential energy of a generic magnetic charge due to existence of the other charges. In a second place, we give the expressions and its demonstrations of the formulas (17)-(24).

1.-**Determination of the magnetic potential energy** :

(1)

This formula is used in our model for obtaining different equations which are basic for the development of the main concept and method of the paper. It is determined from the Classical Statistics Physics in order to relate the magnetronic potential with the probability of obtaining a magnetic charge within the spin-ice systems.

We start from:

(2) where $g_{i}$ is a magnetic charge localized in i position. If one calculates $\nabla n\left(\overrightarrow{r_{i}}\right)$of equation (2), we have:

=

where and are the gradients of magnetic potentials for a positive and negative charge, respectively, whose absolute values are equals, since the probability for appearing a positive magnetic charge is equal to that of the negative magnetic charge. Therefore, we have the simplified expression:

(2)

Therefore, we have

(3)

The C constant is a real number which can be determined if one consider an edge condition for a critical temperature such as that for the first peaks of the specific heat, or for any critical temperature corresponding to any peak whose structure is clearly defined. Therefore, we have the following conditions: $T=T_{V}$, $n\left(\overrightarrow{r}\right)=n_{0}$ and , therefore is the potential energy for each generic charge in the system when it is at temperature $T_{V}$. From the two latter formulae (2) and (3), we have and consequently,

(4)

This formula allows us to determine the energy of any generic individual charges and the confined dipoles magnetic charges.

**2.- Determination of Thermodynamic potential and its derivatives with respect to T.**

Other capital expressions in this model are those which allows us the Helmholtz free energy in both the plasma state and the boson state. Therefore, we give and explain the mathematical derivations of the Eqs. (18)_(24). We will start from the functions of the free energy of Helmholtz which is defined as:

$$F=-\frac{1}{\beta}\left\{2\sum_{i} \text{l}n\left[1+\text{e}xp\left(-\beta(e'_{i}-\mu')\right)\right]-\sum_{j} \text{l}n\left[1-\text{e}xp\left(-\beta(e_{j}-\mu)\right)\right]\right\}$$

and having in mind Eq. (15) of the main text of article and considering the energy of the individual energy of respective components, , we have:

From these latter equations, we obtain: and defining the following physical variables $b'=\Sigma_{0}+\frac{\Delta^{2}}{(<e_{j}>^{2}+\Delta^{2})^{1/2}}-\mu$, $b=\Sigma_{0}-\frac{\Delta^{2}}{|M'V_{0}|}-\mu$, and we obtain:

$F_{BEC}=N'\frac{K_{B}T}{n_{0}}\int_{0}^{n_{0}} \ln\left|1-\left(\frac{n}{n_{0}}\right)^{2n}e^{-a2n}e^{-b\beta}\right|dn,=N'\frac{K_{B}T}{2n_{0}}\int_{0}^{2n_{0}} \text{l}n\left|1-\left(\frac{t}{2n_{0}}\right)^{t}e^{-at}e^{-b\beta}\right|dt$ (5)

where for obtaining the right-hand side of equality, we have included the variable change $2n=t$. This being the corresponding Helmholtz free energy of the Bose-Einstein condensate. From Eq. (5), we determine in a first derivative with respect to temperature, the expression of entropy and from the second derivative the specific heat:

$$S_{BEC}(T)=-\frac{dF_{BEC}}{dT}=-\frac{d}{dT}\left[N'\frac{K_{B}T}{2n_{0}}\int_{0}^{2n_{0}} \text{l}n\left|1-\left(\frac{t}{2n_{0}}\right)^{t}e^{-at}e^{-b\beta}\right|dt\right]=K_{B}\left[\frac{-N}{14n_{0}}\left(I_{1}-b\beta e^{-b\beta}I_{2}\right)\right]$$

And from this expression of the global system, the entropy per each of the $N$ vertexes, considering all vertexes of the crystalline structure with the same probability for suffering a spin-flip, we have:

$s_{BEC}(T)=-\frac{K_{B}}{14n_{0}}\left(I_{1}-b\beta e^{-b\beta}I_{2}\right)$ (6)

and from the second derivative, $c_{BEC}(T)=-\frac{1}{N'}\frac{d^{2}F_{BEC}}{dT^{2}}$ the specific heat:

$c_{BEC}(T)=\frac{K_{B}}{2n_{0}}\left[\frac{b^{2}\beta^{2}e^{-b\beta}}{2n_{0}}\left(I_{2}+e^{-b\beta}I_{3}\right)\right]$ (7)

These two physical magnitudes whose units are the same, depends on the three integrals whose calculation is only possible from a computational calculation whose expressions are:

$I_{1}=\int_{0}^{2n_{0}} \ln\left|1-\left(\frac{t}{2n_{0}}\right)^{t}e^{-at}e^{b\beta}\right|dt$ (8)

$I_{2}=\int_{0}^{2n_{0}} \frac{\left(\frac{t}{2n_{0}}\right)^{t}e^{-at}}{\left|1-\left(\frac{t}{2n_{0}}\right)^{t}e^{-at}e^{b\beta}\right|}dt$ (9)

$I_{3}=\int_{0}^{2n_{0}} \frac{\left(\frac{t}{2n_{0}}\right)^{2t}e^{-2at}}{\left(1-\left(\frac{t}{2n_{0}}\right)^{t}e^{-at}e^{b\beta}\right)^{2}}dt$ (10)

From Eqs. (5), (6) and (9), one can determine the internal energy of the global boson state independently if this is either a BEC state or not.

Concerning the plasma state and by a procedure similar to that carried out in the global bosonic state, we have.With a similar variable change $1-2n=t$, we obtain, the Helmholtz free energy of the plasma state: With a similar variable change $1-2n=t$, we obtain, the Helmholtz free energy of the plasma state:

$F_{Plasma}=-2N'\frac{K_{B}T}{m_{0}}\int_{0}^{m_{0}} \ln\left(1+\left(\frac{n}{n_{0}}\right)^{1-2n}e^{-a(1-2n)}e^{-b'\beta}\right)dn=-N'\frac{K_{B}T}{m_{0}}\int_{1-2m_{0}}^{1} \text{l}n\left[1+\left(\frac{1-t}{2n_{0}}\right)^{t}e^{-at}e^{-b'\beta}\right]dt$ **(11)**

This being the corresponding Helmholtz free energy of the magnetic plasma state condensate. This thermodynamic potential allows us to determine the entropy by magnetic charge either it has negative or positive charge and the specific heat whose expressions are:

$\begin{matrix} S_{Plasma}(T)=-\frac{dF_{Plasma}}{dT}=\frac{d}{dT}\left[N'\frac{K_{B}T}{2n_{0}}\int_{1-2m_{0}}^{1} \ln\left|1+\left(\frac{1-t}{2n_{0}}\right)^{t}e^{-at}e^{-b'\beta}\right|dt\right]=\frac{N'K_{B}}{m_{0}}\left[I_{4}+b'\beta e^{-b'\beta}I_{5}\right] \\ s_{Plasma}=\frac{K_{B}}{m_{0}}\left[I_{4}+b'\beta e^{-b'\beta}I_{5}\right] \end{matrix}$ (12)

$c_{Plasma}(T)=\frac{K_{B}}{m_{0}}\left[\frac{b^{2}\beta^{2}e^{-b\beta}}{2n_{0}}\left(I_{5}-e^{-b'\beta}I_{6}\right)\right]$ (13)

The integrals which appear in Eqs. (12) and (13) only can computationally be determined and whose expression are the following

$I_{4}=\int_{1-2m_{0}}^{1} \ln\left[1+\left(\frac{1-t}{2n_{0}}\right)^{t}e^{-at}e^{b\beta}\right]dt$ (14)

$I_{5}=\int_{1-2m_{0}}^{1} \frac{\left(\frac{1-t}{2n_{0}}\right)^{t}e^{-at}}{1+\left(\frac{1-t}{2n_{0}}\right)^{t}e^{-at}e^{b'\beta}}dt$ (15)

$I_{6}=\int_{1-2m_{0}}^{1} \frac{\left(\frac{1-t}{2n_{0}}\right)^{2t}e^{-2at}}{\left(1+\left(\frac{1-t}{2n_{0}}\right)^{t}e^{-at}e^{b'\beta}\right)^{2}}dt$ (16)

In a similar way to the global boson case, we can deduce from Eqs. (11), (14) and (16), the internal energy for the global plasma state of the spin-ice systems. There is a difference between the expressions (10)-(12) corresponding to bosonic global system and those which correspond to the plasma state, the expressions (13)-(15). This difference is the possibility of divergence within the equations (10)-(12) and consequently the possibility of appearance of a first order thermodynamic phase transition in the bosonic phase in front of the impossibility of these divergences in the corresponding integrals of the plasma state. This will be reflected in the result of the specific heat. On the other hand, we can determine the internal energy both the boson condensate and the magnetic plasma state from Eqs. (5) and (6) for the internal energy of the boson condensate and with equations (11) and (12) for the magnetic plasma state.
